# The dignity of the human corpse in forensic medicine

**DOI:** 10.1007/s00414-021-02534-x

**Published:** 2021-03-11

**Authors:** Clara-Sophie Schwarz, Nikolai Münch, Johannes Müller-Salo, Stefan Kramer, Cleo Walz, Tanja Germerott

**Affiliations:** 1 Institute of Forensic Medicine, Johannes Gutenberg University Medical Center, Am Pulverturm 3, 55131 Mainz, Germany; 2 Institute for the History, Philosophy and Ethics of Medicine, Johannes Gutenberg University Medical Center, Mainz, Germany; 3 Institute of Philosophy, Gottfried Wilhelm Leibniz University, Hannover, Germany

**Keywords:** Autopsy, Ethics, Dignity, Forensic medicine, Human corpse

## Abstract

Working with the dead is a very specific kind of work. Although a dignified handling of the corpses is demanded by the legislator and by the general public, neither the legal status of the corpse is undisputed nor is it obvious what a dignified handling of the deceased should consist of. In our hypothesis generating pilot study, we asked which concrete considerations are involved in daily practice of forensic specialists. We used an online questionnaire (invitations via e-mail) consisting of questions with single choice, multiple choice, and free text entries. The answers to single or multiple choice questions were displayed in pivot tables. The data was thus summarized, viewed, descriptively analyzed, and displayed together with the free text answers. 84.54% of the physicians and 100% of the autopsy assistants stated that considerations concerning the dignity of the deceased should play a role in daily autopsy practice. 45.87% stated that the conditions surrounding the autopsy need improvement to be ethically suitable. The analysis of the survey’s results was based on Robert Audi’s ethics, according to which three aspects need to be lightened in order to evaluate the conduct of a person morally: the actions, the motivation, and the way in which the actions are carried out. This systematization helps to identify the need for improvement and to make the vague demands for a dignified handling of corpses more concrete.

## Introduction

Autopsies are still the gold standard in diagnosing the cause of death, but they are invasive procedures on the human corpses. In practice, one often experiences that lay people present, for example students or police officers, perceive autopsies as irritating. For many people, the autopsy procedure is a taboo break, which can only be justified by the expected benefits of the autopsy. Various German burial laws require that when dealing with human corpses “the dignity of the deceased and the moral sensibilities of the general public are not violated” (Bavaria) or that “the reverence of the dead person” must be maintained (Berlin). Yet, the legal status of human corpses is not beyond dispute [1, 2]. While some argue that the human corpse is a thing in legal sense, others claim that (at least some) personality rights apply also to the dead. This is, as Ellen Stroud puts it, the “central puzzle of the law of the dead”: “The human corpse is a thing, a material object—a messy, maybe dangerous, perhaps valuable, often useful, and always tangible thing—and the law has much to say about such things. But the dead human body is also something very different: It is also my father, and my friend, perhaps my child, and some day, me. For even the most secular among us, a dead human body is at the least a very peculiar and particular kind of thing.” [3]. Yet, even if one can agree that a dead human body is a very particular kind of thing, what it means to treat a dead body in a dignified way or respect the dignity of a dead body is not at all clear. Even in regard to the living the term “dignity” was and still is in center of debates of philosophers and ethicist [4] and law scholars alike [1]. Although “dignity” plays a major role in medical ethics and bioethics [5]—especially in Germany [6]—and is embedded in many international policy documents which were adopted by intergovernmental bodies such as the UN, UNESCO, and the Council of Europe, it is nowhere precisely defined [1, 4]. This vagueness has led to the claim that dignity is a “useless concept […] and can be eliminated without any loss of content” [7] as it “contains nothing that isn’t more clearly conveyed by the widely accepted principle of respect for persons” and their autonomy [8]. This argument was vigorously attacked and many authors tried to develop a differentiated and multi-faceted notion of dignity to show that this term is more than just respect for the autonomy of persons [4, 5, 9, 10] and that its vagueness does not render “dignity” unuseful as many principles (in contrast to concrete rules) in ethics and law are general in the same sense [4]. Interestingly, many of those authors who defend the usefulness of dignity as a general principle of ethics and/or law also give examples where dignity functions as a principle that indicates which kinds of treatment of human corpses are unethical. So, they characterize dignity as a normative principle that (usually *ex negativo*) guides handlings and dealings with living and dead people alike [8, 9, 10]. Here “dignity” should indicate what dealings with human corpses are unethical based on the assumption that these corpses are—as above cited—a somehow peculiar thing. To spell out why “using the head of a dead person as a football” is not appropriate, Charles Foster is convinced, “nothing but dignity will do” [10]. But if it is hard to define what the dignity of a living person implies, it gets even harder with human corpses as here even the question if and how you can harm a dead person is disputed [11–13].

Given this state of discussion in law, ethics, and philosophy about the status of human corpses and the understanding of terms to describe it, it is not surprising that the laws do not contain any guidelines as to what a dignified treatment of the human corpse should look like in concrete terms: there is no blueprint. Nevertheless, the wording of the legal texts can be seen as an indication that the general public has certain expectations on people who handle human corpses. Many autopsy specialists call for higher autopsy rates in order to improve legal security and to achieve reliable statistics on causes of death. To achieve these goals, not only an increase in financial resources is obviously needed. It is also necessary to address the demands for dignified handling of human corpses in order to strengthen confidence in the medical staff involved in autopsies. The topic is also likely to be relevant for the medical self-image and “mental hygiene” of the autopsy specialists. Thus, Stefenelli [14] notes that in the past, but above all in the present, the possibility of getting used to handling human corpses without blunting has been pointed out. Experiences of professionals dealing with the dead, however, indicated that this could only be achieved after a thorough reappraisal of one’s own relationship to the human corpse.

## Material and methods

The presented work is a hypothesis generating pilot study. The aim is to find out how the “dignity and respect for the deceased” required by German burial laws is actually reflected in autopsy practice and how it should be reflected from the autopsy specialists’ point of view.

In autumn of 2019, autopsy specialists at institutes of forensic medicine in Germany were asked to fill out a questionnaire. For this purpose, the list of institutes of forensic medicine on the homepage of the German Society for Legal Medicine was used as a basis. Subsequently, the e-mail addresses of the staff were researched on the homepages of the institutions. If the e-mail addresses of employees were not available, chief physicians were contacted with the request to forward the e-mail to their employees. In the e-mails, the addressees were informed about the purpose of the study. They were asked to fill out a questionnaire processed by “SoSci Survey” (German provider of web surveys; free of charge for the academic sector). The questionnaire consisted of questions with single choice, multiple choice, and free text entries and included information on gender, age, autopsy experience, professional position, and practices in the autopsy room.

The answers of the participants were sent through an encrypted SSL connection. The IP addresses of the participants were not stored, so that personal information cannot be associated with individual survey results or the particular institution.

After the survey was completed, the answers to single or multiple choice questions were displayed in pivot tables. The data was thus summarized, viewed, descriptively analyzed, and displayed together with the free text answers.

## Results

A return rate cannot be determined due to the chosen procedure of sending e-mails to chief physicians for forwarding to employees. A total of 109 participants answered the questionnaire in full. These are 58 women (53.21%) and 51 men (46.79%). Almost half of the participants were in their third decade of life at the time of the survey. Ninety-seven participants are physicians (88.99%), 12 autopsy assistants (11.01%).

84.54% of the physicians and 100% of the autopsy assistants stated that, in their view, considerations of the dignity of the deceased should play a role in daily autopsy practice. It was noticeable that only 4 out of 36 (10.53%) residents (“Assistenzärzte”) and 2 out of 26 specialists (“Fachärzte”) without a management function (7.69%), but 9 out of 26 head physicians (34.62%) were of the opinion that considerations of dignity should not play a role (Table 1).

**Table 1:**
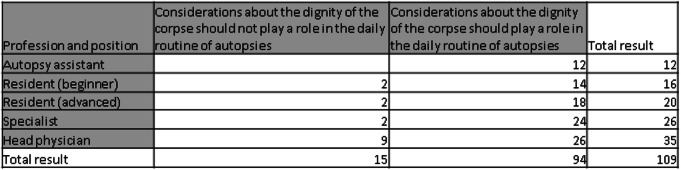
Should considerations about the dignity of the corpse play a role in the daily routine of autopsies?

The participants were also asked about the current situation in their own institutions regarding the dignified handling of bodies (Table 2). It turned out that a total of 13 (11.93%) stated that considerations of dignity should not and indeed do not play a role. 75 (68.81%) declared that dignity should play a role and in fact does play a role. However, the desired state and the actual state differed for 21 persons interviewed. 2 participants (1.83%) stated that dignity plays a role, although in their opinion this is not appropriate. 19 (17.43%) stated that dignity does not play a role, although it should play a role.

**Table 2:**
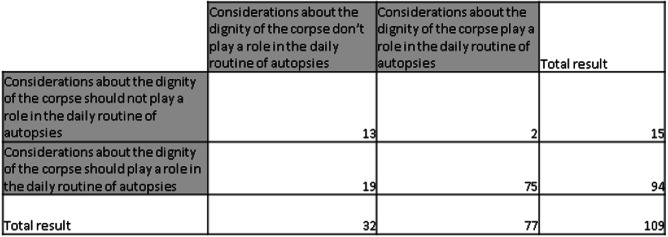
Comparison of the actual state with the desired state regarding dignified handling of the corpse

It was also asked whether the participants would refuse the autopsy of a relative if it was possible. This question was answered in the affirmative by 17 persons (15.60%). Of these 17 persons, 8 (47.06%) stated elsewhere that they did not find the procedures in their own institute worthy of improvement. For now, it remains an open question whether there is distrust of other institutes or whether the procedure of autopsy can generally not be thought of together with the own relatives. In the latter case, the follow-up question arises as to whether there is a very strong professional distancing from death and dying as well as autopsies, or whether one’s own activity is even experienced as not being useful or degrading. Religiousness does not seem to be a decisive factor in this context: only 3 participants who described themselves as religious would prevent the autopsy of a relative. On the other hand, the other 33 religious people would not generally refuse an autopsy of a relative. It is important to mention that at least one person apparently misunderstood the question about the autopsy of a relative, according to the free text. It remains open whether the question was also misunderstood by other participants and the result is therefore distorted.

Those who stated that they wanted to prevent the autopsy of a relative were asked in a free-text question about their reasons. In fact, doubts about the adequate performance of autopsies in foreign institutes were mentioned. In addition, the answers provided evidence that the private individuals maintain a strong distance from their professional life, but also that autopsies are generally considered problematic and degrading. The latter seems irritating, since all of the persons questioned regularly perform autopsies themselves. Finally, it seems challenging to investigate whether a professional activity that is seen as being so problematic can be integrated into a coherent self-image. There were even indications that the sense of autopsies is fundamentally questioned: among other things, it was pointed out that an autopsy does not bring the family member back to life. However, this is undoubtedly true for all deceased persons whose bodies are autopsied—including those whose bodies are autopsied by the participants of the questionnaire.

**Fig. 1:**
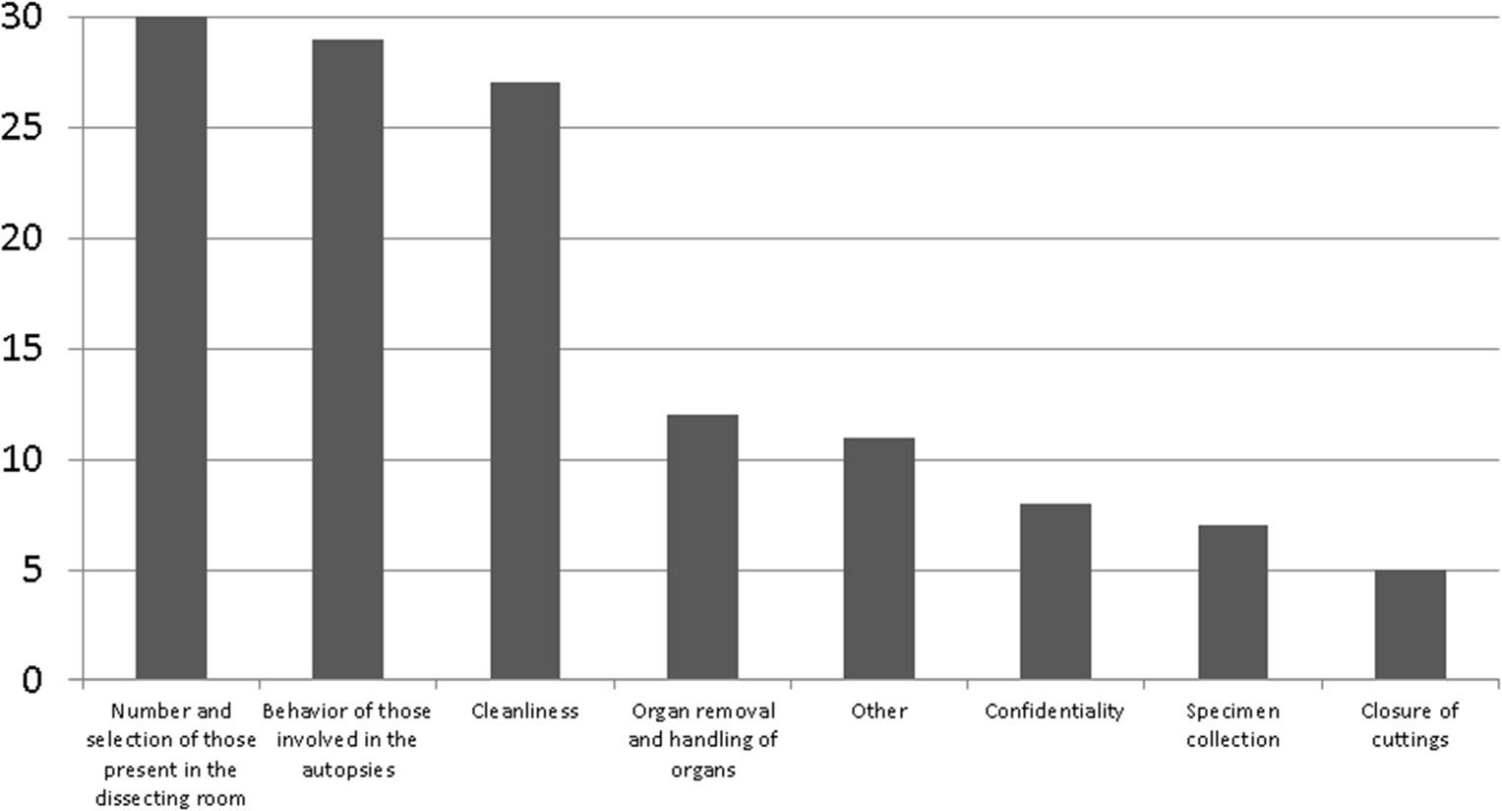
What is perceived as in need of improvement?

**Fig. 2:**
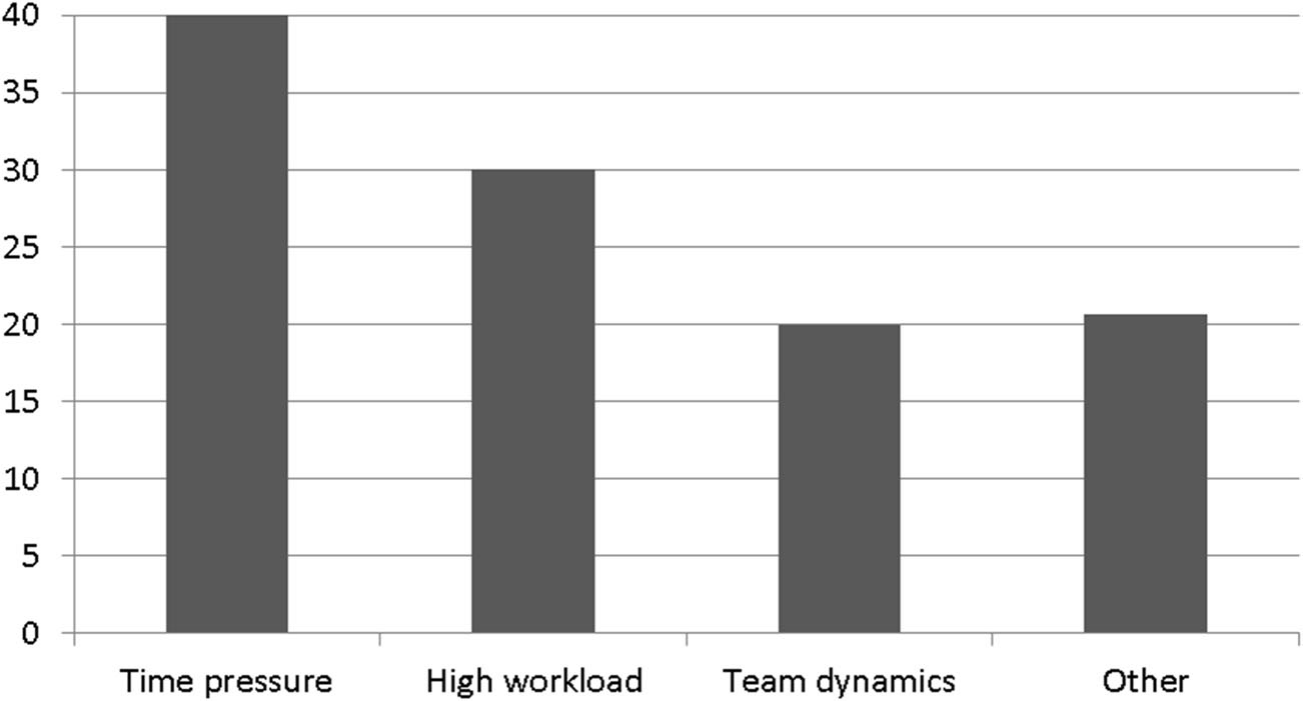
Working conditions interfering with the dignified handling of corpses

Fifty participants (45.87%) claimed that they thought the processes in their institute could be improved (Fig. 1). Even more than half of the respondents, 56 persons (51.38%) stated that certain working conditions interfered with the dignified handling of human corpses in their everyday work (Fig. 2). The most common areas for improvement reported by the residents were the number and selection of people who are present in the dissecting room, by the specialists the behavior of those involved in the autopsies, and by the head physicians the cleanliness. Autopsy assistants most often mentioned the behavior of those involved in the autopsies and cleanliness as factors in need of improvement. In the free text (“Other”), the condition of the rooms was repeatedly criticized as being in need of improvement, as well as the storage and transfer of human corpses. The problem of the (lack of) respect for the will of the deceased was also mentioned, and the behavior of interns such as legal trainees and students was problematized. These spectators sometimes perceived autopsies as disgusting or as a thrill. Personal interests of those involved (e.g., the own scientific career) and economic aspects could also impair the dignified treatment of the dead. The condition of the human corpse themselves (e.g., severe overweight or putrefaction) could make dignified handling difficult. Once again, the problem of spectators was raised several times. In the free text (“Other”), a few individuals questioned the fact that autopsies and dignified handling of human corpses can be thought of together at all.

The participants were also asked whether it should be the responsibility of university-based forensic medicine to work out ethical aspects in dealing with human corpses with medical students and whether ethical aspects should be part of the specialist medical training. The former was affirmed by 83 (76.15%), the latter by 79 (72.48%) persons. It was striking that all 12 participating autopsy assistants answered both questions in the affirmative (Tables 3 and 4).

**Table 3:**
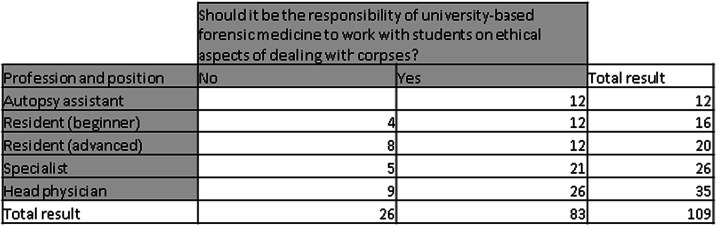
Should it be the responsibility of university-based forensic medicine to work with students on ethical aspects of dealing with corpses?

**Table 4:**
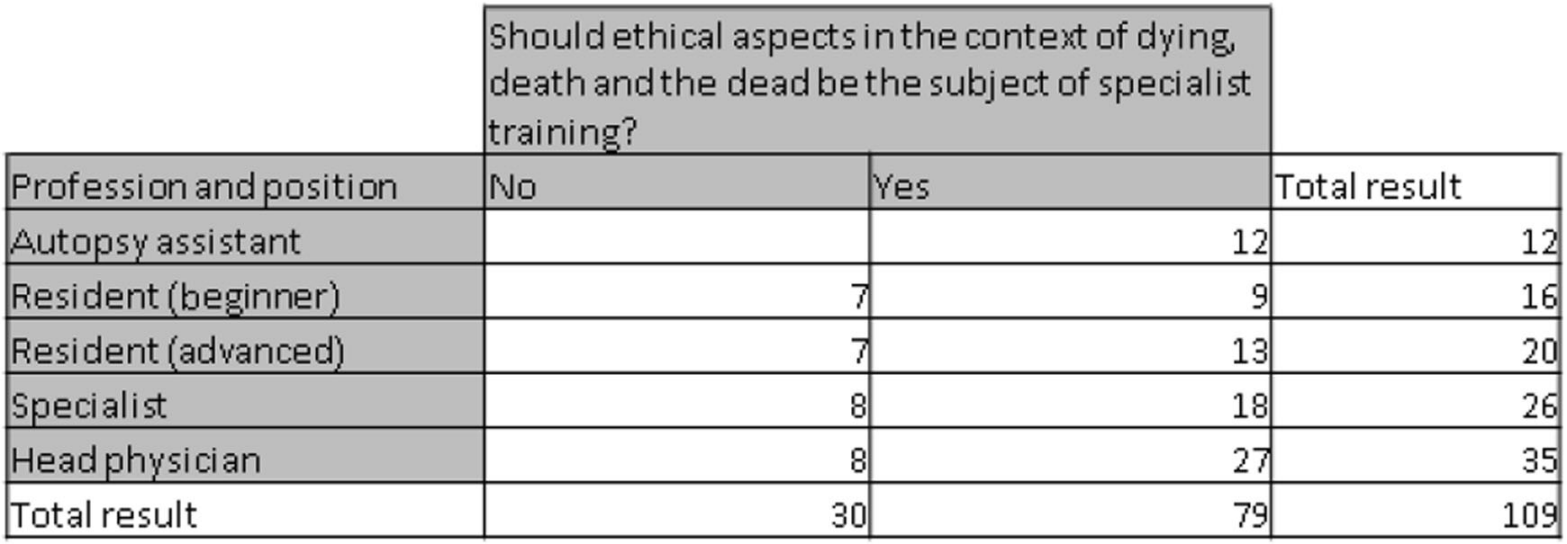
Should ethical aspects in the context of dying, death and the dead be the subject of specialist training?

## Discussion

The survey results indicate that the majority of physicians and autopsy assistants consider it appropriate to critically reflect on the handling of the human corpse. Some individuals, however, did not share the view that considerations about the dignified handling of human corpses should be part of forensic pathology. It is possible that dealing with such questions may be seen as endangering the neutral expert view, or that personal convictions regarding the ontological status of the human corpse may be underlying. Nevertheless, some individual answers also indicate that autopsies are not considered useful or that the performance of autopsies is perceived as problematic regarding the dignity of the deceased per se.

Holczabek [15] answers the question why a doctor chooses to spend his professional life mainly with the dead by referring to the realization that this doctor is dealing with irreplaceable, important tasks. It is plausible, he says, that by concentrating on the documentation and interpretation of medical findings, the terrible is receding. The focus on the responsible professional task characterizes the relationship of a forensic pathologist to the human corpse. According to Holczabek, it is probably understandable for everyone that the human corpse itself is pushed into the background, but the deceased person is granted the entire knowledge and skills of the doctor.

On the one hand, this argumentation is congruent with philosophical positions that ground a dignified or respectful dealing of human corpses in the interests of the deceased person [e.g., 13]. On the other hand, it is noticeable that physicians and autopsy assistants primarily regard certain working conditions as interference in the dignified handling of human corpses. The number and selection of people present in the dissecting room, their behavior, the cleanliness, but also time pressure and high workload are seen as factors that disturb the professional care of the physician in the best interest of the deceased person which is perceived as an undignified handling of the human corpse. Primarily these disturbing factors possibly affect a handling *lege artis* which in turn does not interfere with the dignity of the human corpse. Based on this observation, it seems promising to relate the concept of dignified treatment closely to the concept of appropriate, respectful conduct. While ethics usually focuses on (individual) actions and seeks to evaluate them from a moral point of view, the term “conduct” serves to broaden the perspective. And such an expansion seems necessary in order to cover the survey’s results adequately.

In recent years, Robert Audi has developed an ethics of conduct that locates itself in the tradition of Immanuel Kant. It is, therefore, particularly suitable for analyzing the survey’s results, as Kant’s philosophy has a decisive impact on the general as well as the legal and theoretical understanding of the concept of dignity in German-speaking countries. According to Audi, three aspects must be taken into account in order to evaluate the conduct of a person morally: The actions, the motivation, i.e., the motives underlying the actions, and finally the way in which the actions are carried out, i.e., the manner of acting [16: e.g., 53f., 64].

Let us first consider the aspect of actions. As already mentioned, the results of the survey, especially the answers given to the open questions, allow us to conclude that many participants connected respect for dignity with actions that meet the professional standards of forensic medicine, that are performed *lege artis*. If it is assumed that forensic medical autopsies—in contrast to what few of those questioned seem to assume—are in principle compatible with the preservation of dignity, it is consistent to demand that only such actions are carried out that correspond to the current state of knowledge in the field and, therefore, fulfill standards of best practice.

A look at the component of motivation is revealing. According to Audi, motivational aspects are important because they shed light on the agent’s character: does she use others merely as means to her goals or is she prepared to show genuine interest and respect? [16: 30, 59, 101ff., 143]. The answers from the survey make it clear that in the eyes of some of the survey’s participants, adequate motivation is also important in assessing the question whether or not a human corpse is treated with dignity during autopsy. This becomes particularly obvious when participants address the questionable role of certain personal interests, for example in one’s own scientific career, or the importance of economic aspects. Such cases can be described as cases of morally problematic motivation, since the agents are not motivated by a genuine interest in the fate of the deceased person but see the corpse as a pure means to an external end—for example, as a means of increasing their own scientific output or as a means of improving the institution’s economic situation. Some of the answers in the survey also raise questions that can be assigned to the aspect of motivation with regard to the observing participants: are the participants motivated by a genuine interest in the practice of autopsy, in fostering their medical knowledge or in continuing their investigations? Or is undirected curiosity and thrill what motivates them to participate?

Perhaps most important is the third component Audi identifies, the aspect of manner: “In practice, there are limits to the number of ways we can control the manner of our actions; but for a huge range of act-types that we can consider realizing […] *how* we should do the thing in question is morally significant. […] In principle, no matter how complex an action is, there will be more than one way it can be performed.” [16: 55]. The state *lege artis* of forensic medicine determines which actions are to be performed during the autopsy. However, these actions can be performed in a variety of ways—and the answers from the survey clearly indicate that the manner is crucial in determining whether the corpse’s dignity is preserved or not during autopsy.

Questions of manner are important with regard to the setting chosen for autopsy. The same actions can be performed in a clean and bright room or in a dubious surrounding that is more reminiscent of a poorly maintained storage room. The same holds true with regard to the handling of the corpse: during the transport of the corpse, do those involved treat it as if it were a bearer of dignity that deserves respect—or do they treat it like a bulky, heavy load that has to be transported to its destination as quickly as possible? Finally, the autopsy itself: is it performed quickly and with purely technical precision? Or are small actions and gestures integrated into the process, which—such as washing the hair after the procedure is finished—can be interpreted as an expression of respect and remind all participants that they are dealing with a deceased person? The answers in the survey clearly indicate that these manner-related questions are crucial for the autopsy’s overall moral evaluation.

As this brief overview shows, it makes sense to assign the criticism expressed in the survey to different components of conduct. This systematization is important not only in terms of moral evaluation, but also with regard to practical improvements. For each aspect perceived as problematic, it can be asked whether a change in the external circumstances, the introduction of new rules and procedures, or a change in the internal attitude of those involved is necessary for reorganizing the procedures in a way acceptable from a moral point of view.

## Conclusion and prospects for future research

There are good reasons to deal with ethical aspects of everyday professional life as a practicing forensic physician. A dignified handling of human corpses is demanded by legal regulations and expected by the public alike. But what such a dignified handling requires in the concrete practice of forensic medicine is not spelled out in law nor in medical ethics. In the study carried out here, it could be shown that, from the perspective of forensic physicians and autopsy assistants, a dignified handling of human corpses is a central topic in daily practice of forensic medicine. Many of those practitioners believe that certain working conditions interfere with the dignified handling of human corpses in their everyday work, whereby primarily external conditions were judged to impede dignified handling of corpses. The results of the survey are generally suitable for reflecting on the question of dignified handling of corpses on a practical level. It remains a task to specify further how daily handling and routine—especially regarding those identified external factors—should be structured and shaped to meet the demands and expectations of all stakeholders for a dignified handling of human corpses—forensic medicine practitioners, patients, relatives, and the wider public.

Furthermore, since the perception of spectators who are only exceptionally present at an autopsy may differ from that of the more experienced, a survey of such persons appears to be a promising addition to the previous research results. A comparison of both groups could also provide enlightening results. In this context, the psychological aspect of regular confrontation with the dead may be of interest for the professional self-image of forensic scientists and could be a future object of research.
